# Bridge of Rainbow: Association Between Internet-Based Social Media Use and Homosexuality Inclusion in China

**DOI:** 10.3389/fpsyg.2022.882206

**Published:** 2022-06-16

**Authors:** Yao Jiang, Fan Yang

**Affiliations:** ^1^Zhou Enlai School of Government, Nankai University, Tianjin, China; ^2^Department of Labor and Social Security, School of Public Administration, Sichuan University, Chengdu, China

**Keywords:** internet-based social media, information technology development, inclusion of LGBT, sexual psychology, China

## Abstract

By using the nationally representative dataset of China Labor-force Dynamics Survey, this paper explored the relationship between internet-based social media use and Chinese people's homosexuality inclusion. Addressing endogeneity by using an instrumental variable approach, the results of instrumental variable-ordered probit model indicated that individuals' internet-based social media use had a positive and significant association with their homosexuality inclusion. Furthermore, the heterogeneity analysis revealed that the heterogeneous effects of internet-based social media use on homosexuality inclusion caused by income, gender, and region. The homosexuality inclusion of respondents with higher income, respondents of female gender, and respondents located in eastern region of China was found to be more evidently associated with internet-based social media use. The functional mechanism analysis suggested that the number of respondents' LGBT friends mediated the overall relationship between internet-based social media use and homosexuality inclusion. The robustness check showed that the results were robust cross different models. The findings in this paper provide new evidence that the effect of information technology development on individual perception and behavior in Chinese context.

## Introduction

LGBT (lesbian, gay, bisexual, and transgender) population were once a hidden group in Chinese mainstream domains, and the discussion of related topics was rather ambiguous and sensitive (Wang et al., 2019). With the modernization of China, online social interactions among individuals have been tremendously promoted. By 2021, the number of internet users in China reached 1.032 billion (China Internet Network Information Center, 2022). Meanwhile, many discussions and representations on homosexuality have arisen on internet-based social media (e.g., Weibo, WeChat, and Zhihu). As of 14 April 2022, on Weibo (Chinese YouTube), there were 900 thousand posts with the topic of “I'm Lesbian”, and the amount of reading was 620 million. The topic of “Topics between gay men” had 172 thousand posts and 45 million readings. Moreover, on Zhihu (Chinese internet high quality Q & A community), the topic of “homosexuality” was followed with interest by 314 thousand people, and 57 thousand related questions were put forward. The content covered but not limited to affectional problems, display of lifestyles, same-sex marriage, and popularization of science on homosexuality.

Therefore, the objectives of this study were to understand the relationship between internet-based social media use and Chinese public's homosexuality inclusion, and to explore the influential mechanism through which the internet-based social media use can correlate with individuals' homosexuality inclusion.

The most popular gay dating application in China, Blued, had 40 million registered users by the end of 2018 (Blued, 2018). In contrast to the high visibility of the homosexual individuals on internet-based social media, in the practical public spheres, they remain relative closeted (Lin et al., 2016). Some studies have pointed out the invisibility of the homosexual individuals in the real world can be attributed to suppression by the state, which leads to the public's homophobic sentiments in China (Adamczyk and Cheng, 2015; Cheng et al., 2016; Hu and Li, 2019). In fact, the invisibility of homosexual individuals in Chinese mainstream domains and people's homophobic sentiments are products of politics and traditional values. From the perspective of politics, since the foundation of the People's Republic of China in 1949, homosexuality was seen as an inappropriate ideology (Li et al., 2010). The behavior of men who have sex with men was classified as hooliganism and punished by Chinese Criminal Law before 1997 (Xie and Peng, 2017). Furthermore, homosexuality was classified as a mental disorder in the Chinese Classification and Diagnostic Criteria of Mental Disorders before 2001 (Xie and Peng, 2017).

From the perspective of traditional values, in ancient imperial China, homosexuality was a privilege of male ruling classes who enjoyed the company of male concubines (Wu, 2004). Although the homosexuality of the male ruling class is never considered a “disease” in ancient China, the vital filial pieties of raising offspring and continuing one's family line are common traditional practices of the ordinary people that are maintained today (Burki, 2017). Homosexuality apparently runs counter to those crucial traditional values, and it is therefore noted as a phenomenon of social transgression (Feng et al., 2012; Chow et al., 2017).

Based on the above two aspects of politics and traditional values, the homosexual individuals experience multiple pressures from society, families, and their own hearts, and most of them are deeply closeted in the real world. Some of them even use “sham marriages” (a marriage of formality or convenience for one or both married partners) to hide their true sexual orientations, avoid stigma, or continue their family line (Choi and Luo, 2016).

Regarding the association between internet-based social media use and Chinese people's homosexuality inclusion, conclusions have been mixed in the literature. By employing the ordinary least square (OLS) regression, Lin et al. (2016) focused on the attitudes of 494 college students in Beijing and Xiamen, China, toward homosexuality, and they argued that the college students generally held accepting attitudes and their greater exposure to internet-based media led to greater homosexuality inclusion (Lin et al., 2016). Chi and Hawk (2016) used the data of 2,644 Chinese university students and employed OLS to find that a higher frequency of internet use was correlated with a more positive attitude toward the same-sex behavior. Furthermore, Zheng et al. (2022) employed the natural language processing and machine learning techniques to explore Weibo users' attitudes toward lesbian or gay, and the results suggested that these users had generally positive attitudes regarding the rights of lesbians and gays.

However, Tu and Lee (2014) investigated the attitudes of 226 college students who received education and lived in Beijing or Shanghai, China, toward the gay and lesbian population, and they proposed that the usage of Chinese media reinforced the college students' higher level of negative homosexuality stereotypes. Tu and Lee (2014) concluded that the negative effect of Chinese media usage on college students' attitudes toward homosexuality could mainly be attributed to efficiently maintained traditional filial pieties.

Overall, though the above-mentioned literature provides a guideline, the crucial role of internet-based social media in users' attitudes toward homosexuality in China is still unknown. We decided to fill three research gaps in this study. First, the samples of most of the existing literature, such as college students in developed cities (e.g., Beijing or Xiamen) of China, were rather non-representative of the country as a whole. Though college students' attitudes toward homosexuality may aid the prediction of social attitudes in the future, they can only represent the attitudes of a part of the public who are well-educated and speculative. In this study, we used a nationally representative dataset of China Labor-force Dynamics Survey 2018 (CLDS 2018) to understand the Chinese public's homosexuality inclusion. We expanded the list of potential influencing factors of homosexuality inclusion in the Chinese context and considered the social effects of technological information development in the field of sexual psychology.

Second, few attempts were made to explore the potential mediator through which the internet-based social media use can correlate with individual attitudes toward homosexuality, and the heterogenous effect of internet-based social media use on homosexuality inclusion. In this study, we explored the mechanism through which internet-based social media use can be associated with individual homosexuality inclusion. We also presented heterogeneities in the effects of internet-based social media use on homosexuality inclusion at the different levels of income, gender, and region.

Third, the relationship between people's internet-based social media use and their attitudes toward homosexuality needs to be discussed more carefully. Specifically, the OLS models were frequently used in the literature. It indicates that the endogeneity problem between internet-based social media use and homosexuality inclusion, involving the unobserved confounding factors and measurement errors, is not addressed. In this study, we adopted the instrumental variable (IV) approach to address endogeneity to explore the relationship between internet-based social media use and homosexuality inclusion in Chinese setting.

The remainder of this paper is organized as follows. In Section Theoretical Framework and Hypothesis Development, we present our theoretical framework and the research hypotheses. Section Methods provides our data, measurements, and data analysis strategy. Section Results presents the empirical results, and Section Discussion incorporates discussions of the results. Section Conclusion presents our conclusions.

## Theoretical Framework and Hypothesis Development

### Internet-Based Social Media Use and Homosexuality Inclusion

The emerging internet-based social media surpasses traditional media, provides freedoms for discussing homosexuality-related topics, and a reference group that holds accepting attitudes toward homosexuality. In China, the homosexuality-related topics are generally banned from traditional mainstream media outlets (e.g., TV, newspaper, and movies); thus, the constructive information for sexuality identification is not easily to be obtained from traditional media (Tu and Lee, 2014; Shao, 2018). However, internet-based social media have attributes of fragmentation, accessibility, affordability, and anonymity, which provides freedoms for discussing homosexuality-related topics (Wang et al., 2018; Liu et al., 2020). Therefore, compared with the traditional mainstream media which generally bans the topics of homosexuality, internet-based social media are not only venues for homosexuality related self-expression, equal right promotion, and community building, but also provide its users with a reference group that holds accepting attitudes toward homosexuality (Wu et al., 2018; Liang et al., 2022). These accepting attitudes can be seen in, for instance, scientific studies on homosexuality posted on internet-based social media that state that homosexuality is just a kind of sex orientation rather than a disease and discussions concerning the affection, life, and harmonious families of LGBT individuals (Zhang, 1994).

More important, internet-based social media provide a reciprocal process that involves two-way conversations between the information receivers and information providers, which offers opportunities for public to reflect on and question their prior acceptance of conventions (Stroud, 2007; Goodman and Moradi, 2008). Unlike traditional mainstream media, the information receivers of internet-based social media do not merely passively receive but actively digest information based on their ascribed value systems (Hu and Li, 2019). For example, in Weibo, the audience can interact with the content creators and other audience members. Internet-based social media can promote the free flow of information, which can complement or controvert audiences' or creators' attitudes (Hu and Li, 2019). The reciprocal process provided by internet-based social media may make individuals reflect on and question their prior accepted conventions, as well as alter attitudes regarding homosexuality. Thus, in the process of interactions with a social reference group who holds an acceptant attitude toward homosexuality, one's original attitudes regarding homosexuality may be altered and their inclusion may be enhanced. Based the above discussion, we propose our first hypothesis.

**Hypothesis 1**. A higher frequent usage of internet-based social media predicts a higher homosexuality inclusion.

### Social Network and Number of LGBT Friends

The usage of internet-based social media facilities the establishment of a diversified social network, and a more diversified social network may allow for larger possibility of individuals to make friends with LGBT population. Technological information development and the appearance of internet-based social media have dismantled the restrictions of kinship and geography on social interactions, thus leading to the construction of social networks with attributes of high differentials in gender, age, sexual orientation, income, and education (Bruning et al., 2020; Liu et al., 2020; Li and Gao, 2021). Moreover, in real world, LGBT population in China are relative closeted; however, in contrast they have higher level of visibility in cyberspace. Thus, individuals who are frequently exposed to internet-based social media tend to have more heterogeneous and extensive social networks, and have been suggested to have greater opportunities to know about and interact with LGBT population (Li and Kent, 2021).

The usage of internet-based social media may enable more understanding and inclusion for the homosexuality through increasing the number of LGBT friends. A study on Eastern cultural backgrounds investigated the influence of the experience of interacting and contacting gay individuals on people's attitudes of inclusion toward homosexuality, and the results showed that the experience of interacting and contacting gay individuals positively and significantly impacts people's attitudes of inclusion toward homosexuality by deconstructing previous misleading attitudes (Yen et al., 2007). Based on the above discussions, we propose the second hypothesis.

**Hypothesis 2**. The number of LGBT friends can mediate the relationship between individual usage of internet-based social media and homosexuality inclusion.

## Methods

### Data

We employed the CLDS 2018 survey dataset to test the proposed hypotheses (Yang and Jiang, 2020). The data were collected by the Center for Social Science Survey at Sun Yat-sen University. The respondents answered anonymously to fully protect their information. In CLDS 2018, a multi-stage, multi-level probability sampling method proportional to the size of the labor force was adopted (Yang and Jiang, 2022). The main content of the CLDS 2018 included respondent's personal information (e.g., age, education, income, marital status, and social attitudes) and family information (e.g., family size, family income, and family members' characteristics). The dataset included Chinese laborers from 29 provincial administrative units to maintain the national representativeness of the dataset. The samples with invalid answers (including “inapplicable” and “unclear”), missing answers, and obvious logical contradictions answers were cleared. Consequently, 4,037 valid samples remained for use in this paper.

### Measures

Homosexuality inclusion was the core explained variable in this study, and it was measured with the following question: “How is your attitude toward the homosexuality?” Respondents answered, “strongly reject,” “somewhat reject,” “neutral,” “somewhat accept,” or “strongly accept”; these answers were assigned values of 1–5 on a five-point Likert scale, respectively.

Internet-based social media use was our key explanatory variable. It was measured by asking the respondents “How often do you use internet-based social media?” Participants answered, “never,” “rarely,” “occasionally,” or “frequently”, the answers of which were coded as 1–4, respectively.

Shopping online was the alternative explanatory variable for our robustness check. Internet-based social media commonly have buy buttons, and the usage of internet-based social media has a positive and significant association with online shopping-related behaviors (Martínez-López et al., 2020). We measured shopping online as we measured the internet-based social media use variable. The respondents were asked “How often do you shop online?” The answers were coded from 1 (never) to 4 (frequently).

The number of LGBT friends was the mediating variable. We measured it by asking about the number of respondents' LGBT friends. Respondents were asked “How many LGBT friends do you have?” The answer was numerical.

Control variables included individual and family characteristics. Individual characteristics included gender (male = 1; female = 0), education (years of schooling), age (years old), household registration (rural = 1; urban = 0), religion (non-religion = 1; otherwise = 0), logarithm of income, political status (party member = 1; otherwise = 0), marital status (married = 1; otherwise = 0), and social trust toward neighborhood in a same community (very distrustful = 1; somewhat distrustful = 2; normal = 3; somewhat trustful = 4; very trustful = 5) (Sautter et al., 2010; Adamczyk and Cheng, 2015; Berggren and Nilsson, 2016). Family characteristics included the education (years of schooling) of respondent's mother and father (Roi and Mandemakers, 2018).

In addition, regional effects were controlled according to the region where the respondents lived. According to the regional division of China National Bureau of Statistics, the provinces were grouped into three regions: eastern region, central region, and western region (National Bureau of Statistics, 2011). The definitions and descriptive analysis results of all above mentioned variables are shown in Table 1.

**Table 1:** Descriptive analysis.

**Variable**	**Item**					**Percent**	**Freq**.
Homosexuality inclusion	Strongly reject = 1					17.81	719
	Somewhat reject = 2					49.12	1,983
	Neutral = 3					29.63	1,196
	Somewhat accept = 4					3.24	131
	Strongly accept = 5					0.20	8
Internet-based social media use	Never = 1					50.66	2,045
	Rarely = 2					13.57	548
	Occasionally = 3					8.87	358
	Frequently=4					26.90	1,086
Shopping online	Never = 1					53.55	2,162
	Rarely = 2					12.53	506
	Occasionally = 3					18.01	727
	Frequently = 4					15.90	642
Gender	Male = 1					50.85	2,053
Household registration	Rural = 1					72.68	2,934
Religion	Non-religion = 1					91.60	3,698
Political status	Party member = 1					10.48	423
Marital status	Divorced, widowed, and never married = 0					6.42	259
	Married = 1					93.58	3,778
Social trust	Neighborhood in a same community is very distrustful = 1					0.27	11
	Neighborhood in a same community is somewhat distrustful = 2					3.99	161
	Normal = 3					26.55	1,072
	Neighborhood in a same community is somewhat trustful = 4					51.57	2,082
	Neighborhood in a same community is very trustful = 5					17.61	711
Region	Eastern region = 1					50.98	2,058
	Central region = 2					18.48	746
	Western region = 3					30.54	1,233
**Variable**	**Definition**	**Mean**	**S. D**.	**Min**	**Max**	**Quantile 50%**	
Age	Years old	46.550	11.050	17	80	47	
Education		9.715	3.275	6	21	9	
Education of father	Years of schooling	5.414	4.229	0	19	6	
Education of mother		3.540	4.039	0	16	0	
Logarithm of income	Total income in 2017	10.128	1.175	5.704	14.221	10.309	
Number of LGBT friends		0.048	0.250	0	5	0	
Observations		4,037				

### Data Analysis

The explained variable, homosexuality inclusion, represents discrete and ordered data that were assigned values of 1–5 on a five-point Likert scale, and the ordered probit regression can be used to estimate the influence of internet-based social media use on homosexuality inclusion. To verify hypothesis 1, the benchmark ordered probit model used in this study can be written as follows:


(1) $$\begin{array}{l} \;Homosexuality\;inclusion^{*}\;=\\ \alpha_{0}\;+\;internet\;-\;based\;social\;media\;use\;+\;\gamma Z\;+\;\varepsilon \end{array}$$

where Homosexuality inclusion^*^ is the explained variable representing respondents' attitudes toward the homosexuality, α_0_ is the intercept term, *internet-based social media use* is the explanatory variable, respondents' internet-based social media use, *Z* represents a set of control variables, β and γ are the coefficient vectors, and ε is the error term.

In Equation (1), Homosexuality inclusion^*^ cannot be directly observed. We assumed the selection rule of *Homosexuality inclusion* as follows:


(2) $$\begin{array}{l} \;Homosexuality\;inclusion=\\ \{\begin{array}{c} \text{​}=1\;If\;Homosexuality\;inclusion^{*}\leq 1\\ =2\;1<Homosexuality\;inclusion^{*}\leq \mu_{1}\\ =3\;\mu_{1}<Homosexuality\;inclusion^{*}\leq \mu_{2}\\ \cdots \;\cdots \;\\ =J\;Homosexuality\;inclusion^{*}\geq \mu_{J-2} \end{array} \end{array}$$

where μ_1_ < μ_2_ < ⋯ ≤ μ_*J*−2_ are unknown parameters estimated together with β and γ.

Although the ordered probit regression could suggest the association between internet-based social media use and homosexuality inclusion to a certain extent, the possible effect of endogeneity on the reliability of the estimation results were fully considered. To minimize the estimation bias caused by omitted variables, most of factors influencing homosexuality inclusion were controlled, but the endogeneity caused by unobserved confounding factors still could not be ignored. Some prior studies suggested that the formation of homosexuality is closely related to genetic inheritance (Hu et al., 1995; Joslyn and Haider-Markel, 2016; Nathan et al., 2018), while genetic inheritance is a confounding factor that cannot be easily and directly observed through survey data. Therefore, an IV-ordered probit regression method was adopted to overcome the missing control of unobserved confounding factors.

We utilized the average usage frequency of internet-based social media of the other respondents in a same living community as the IV. Theoretically, the average usage frequency of internet-based social media of the other respondents in a same living community is an appropriate IV satisfying the requirements of relevance and exclusion. To keep in touch with others around individuals, the frequency of individuals using the internet-based social media is affected by other individuals in the same living community. However, the average usage frequency of internet-based social media of the other respondents in a same living community is unlikely to affect the homosexual inclusion of the individual directly. As self-selected behavior in residential communities in China is insignificant (Zhao and Yu, 2020), and almost each of living community is inhabited by residents with different socio-economic characteristics together (Chauvin et al., 2016). It is a remarkable feature of Chinese communities (Yang et al., 2022). Therefore, the average usage frequency of internet-based social media of the other respondents in a same living community is a good IV. In fact, it is common to use the average of samples in a certain range as an IV. Studies used this approach to explore the relationship between corruption, taxation, and economic growth, relationship between health and credit constraints (Fisman and Svensson, 2007; Yang et al., 2021a). The results of empirical tests of the IV are presented in Section Results.

Furthermore, the bootstrap approach was employed to test hypotheses 2. The principle of the bootstrapping is that the standard error estimation and confidence interval (CI) calculated based on the assumption of a normal distribution, are imprecise since the indirect effect estimates do not follow a normal distribution (Kong et al., 2015). In the bootstrap approach, the 95% CI is the most important result. If the corresponding 95% CIs of the indirect effect do not overlap with zero, the mediating intermediary effect can be proven.

## Results

### Descriptive Analysis

The results of the descriptive analyses of all variables are provided in Table 1. Out of the respondents, 17.81% of respondents held a “strongly reject” attitude, and nearly half of the respondents (49.12%) showed a “somewhat reject” attitude toward homosexuality. 29.63% of respondents stood neutrally. 3.24% of respondents had an “somewhat accept” attitude regarding homosexuality, and 0.2% held a “strongly accept” attitude. Meanwhile, 50.66% of respondents reported never using internet-based social media, and 53.55% of respondents reported never shopping online. Shopping online showed a roughly similar distribution to internet-based social media use, so employing shopping online as the alternative variable was reasonable and appropriate. Regarding the mediating variable, the maximum value of the respondents' number of LGBT friends was 5, the minimum was 0, and the mean value was 0.048 (SD = 0.250).

Furthermore, the attitudes of respondents toward the homosexuality with different internet-based social media use frequencies were compared by employing the Kruskal–Wallis non-parametric test. The result of the independent sample Kruskal–Wallis test was 100.921, and the corresponding asymptotic significance test value was <0.000. Therefore, respondents with different internet-based social media use frequencies showed significantly diversified attitudes toward homosexuality.

### Results of Benchmark Regression and Instrumental Variables Regression

The results of the ordered probit regression and the IV-ordered probit regression are presented in Table 2. When the individual characteristic variables, family characteristics variables, and regional effects are controlled, the results from the ordered probit model (β = 0.052, *p* < 0.01) and IV-ordered probit model (β = 0.171, *p* < 0.01) both indicate that internet-based social media use is positively and significantly associated with respondents' homosexuality inclusion. This means that the more frequent usage of internet-based social media, the more inclusion for homosexuality. Thus, hypothesis 1 is verified.

**Table 2:** Association between internet-based social media use and homosexuality inclusion (*N* = 4,037).

**Variable**	**Homosexuality inclusion**	
	**Ordered probit**	**IV-ordered probit**
Internet-based social media use	0.052^***^	0.171^***^
	(0.018)	(0.043)
Gender	−0.095^***^	−0.106^***^
	(0.037)	(0.037)
Education	0.032^***^	0.040^***^
	(0.007)	(0.008)
Age	−0.001	−0.006^**^
	(0.002)	(0.003)
Household registration	0.069	0.048
	(0.047)	(0.047)
Religion	0.071	0.084
	(0.062)	(0.062)
Political status	−0.039	−0.014
	(0.061)	(0.061)
Education of father	−0.006	−0.004
	(0.005)	(0.005)
Education of mother	0.011^*^	0.011^*^
	(0.006)	(0.006)
Logarithm of income	0.001	0.027
	(0.018)	(0.020)
Marital status	−0.156^**^	−0.129^*^
	(0.071)	(0.071)
Social trust	−0.028	−0.021
	(0.024)	(0.024)
Regional effect	Yes	Yes
Pseudo *R*^2^	0.008	-
atanhrho_12	-	−0.165^***^
	-	(0.056)
Endogeneity test	Durbin-Wu-Hausman	4.604 (*p* = 0.032)
Weak IV identification test	Cragg-Donald Wald F statistic	171.724
	Kleibergen-Paap rk Wald F statistic	151.937
Under-identification test	Kleibergen-Paap rk LM	144.052 (*p* = 0.000)
Observations	4,037	

*Standard errors in parentheses*;

*** *p < 0.01*,

** *p < 0.05*,

* *p < 0.1*.

The results of the control variables (as measured in Table 2) estimated by the IV-ordered probit model show that gender, education, mother's education, and marital status all have vital effects on Chinese people's homosexuality inclusion. For instance, respondents with higher education are more likely to accept homosexuality (γ = 0.040, *p* < 0.01). Furthermore, mother's education (γ = 0.011, *p* < 0.1) is found to associate with respondents' attitudes toward homosexuality more evidently and significantly than father's education.

The reasonableness of the choice of the average usage frequency of internet-based social media of the other respondents in a same living community as the IV is also reported in Table 2. The result of the Durbin–Wu–Hausman endogeneity test is 4.604 (*p* < 0.05), which significantly rejects the null assumption that internet-based social media use is an exogenous variable. The weak IV identification test presents the Cragg–Donald Wald F statistic and Kleibergen-Paap rk Wald F value of 171.724 and 151.937, respectively, which are significantly higher than the Stock–Yogo weak IV test critical value of 16.38 (10% maximal IV size). Therefore, the IV in this paper is found to be a strong IV. The result of the under-identification test of the Kleibergen–Paap rk LM statistic value is 144.052 (*p* < 0.01), which rejects the null assumption of an under–identification problem and revealed that the IV is closely related to the endogenous variable, internet-based social media use.

### Heterogeneity Analysis

The results of the heterogeneity test of the effects of internet-based social media use on homosexuality inclusion at the different levels of income, gender, and region are presented in Table 3. To test the income heterogeneity of the effects of internet-based social media use on homosexuality inclusion, samples were grouped into two subgroups based on the mean value of income, 38,891 yuan^1^: income > 38,891 yuan and income ≤ 38,891 yuan. The results of income heterogeneity test estimated by IV-ordered probit models are provided in Table 3. The results in Table 3 indicate that compared to the lower income group (income ≤ 38,891 yuan), internet-based social media use has a more significant and positive correlation (β = 0.206, *p* < 0.01) with the homosexuality inclusion of the higher income group (income > 38,891 yuan).

**Table 3:** Heterogeneous effect of internet-based social media use on homosexuality inclusion.

**Variable**	**Homosexuality inclusion (IV**-**ordered probit)**						
	**Income**		**Gender**		**Region**		
	**> 38,891**	**≤38,891**	**Male**	**Female**	**Eastern**	**Central**	**Western**
Internet-based social media use	0.206^***^	0.062	0.105	0.202^***^	0.219^***^	0.141	0.104
	(0.072)	(0.063)	(0.071)	(0.055)	(0.061)	(0.097)	(0.085)
Gender	−0.231^***^	−0.034	-	0.050^***^	−0.174^***^	−0.056	−0.042
	(0.057)	(0.049)		(0.011)	(0.052)	(0.087)	(0.066)
Education	0.033^***^	0.022^*^	0.025^**^	−0.009^**^	0.038^***^	0.037^**^	0.035^**^
	(0.012)	(0.012)	(0.011)	(0.004)	(0.011)	(0.018)	(0.016)
Age	−0.014^***^	0.001	−0.004	0.031	−0.013^***^	−0.005	0.004
	(0.004)	(0.004)	(0.004)	(0.067)	(0.004)	(0.007)	(0.006)
Household registration	−0.088	0.193^***^	0.066	0.045	0.044	−0.088	0.106
	(0.069)	(0.067)	(0.067)	(0.082)	(0.063)	(0.108)	(0.101)
Religion	0.022	0.123	0.112	−0.022	0.060	−0.123	0.199^*^
	(0.099)	(0.081)	(0.095)	(0.100)	(0.083)	(0.167)	(0.116)
Political status	−0.040	0.013	−0.016	−0.005	−0.025	−0.098	0.031
	(0.081)	(0.096)	(0.079)	(0.008)	(0.085)	(0.141)	(0.116)
Education of father	−0.012	−0.001	−0.003	0.013	−0.020^**^	−0.011	0.023^**^
	(0.009)	(0.007)	(0.007)	(0.008)	(0.008)	(0.013)	(0.009)
Education of mother	0.011	0.007	0.008	0.054^**^	0.006	0.016	0.027^**^
	(0.009)	(0.008)	(0.008)	(0.027)	(0.008)	(0.014)	(0.011)
Logarithm of income	0.057	−0.028	−0.010	−0.219^**^	0.088^***^	0.005	−0.031
	(0.054)	(0.029)	(0.031)	(0.102)	(0.031)	(0.041)	(0.034)
Marital status	−0.048	−0.148	−0.037	−0.001	−0.102	−0.384^**^	−0.045
	(0.104)	(0.099)	(0.100)	(0.034)	(0.095)	(0.184)	(0.137)
Social trust	−0.069^*^	0.003	−0.045	0.202^***^	−0.007	−0.093	−0.010
	(0.039)	(0.030)	(0.034)	(0.055)	(0.033)	(0.058)	(0.043)
Regional effect	Yes	Yes	Yes	Yes	-	-	-
atanhrho_12	−0.146^*^	−0.070	−0.058	−0.238^***^	−0.226^***^	−0.064	−0.094
	(0.086)	(0.081)	(0.089)	(0.073)	(0.077)	(0.126)	(0.113)
Observations	1,626	2,411	2,053	1,984	2,058	746	1,233

*Standard errors in parentheses*;

*** *p < 0.01*,

** *p < 0.05*,

* *p < 0.1*.

The results of gendered heterogeneity test show that internet-based social media use has a greater and more positive association (β = 0.202, *p* < 0.01) with homosexuality inclusion among female participants, while the coefficient in the male group is insignificant. Table 3 also reports the regional heterogeneity of the effect of internet-based social media use on homosexuality inclusion. Except for the coefficient of the eastern region group (β = 0.219, *p* < 0.01), the coefficients of the central and western region subgroups are insignificant.

### Robustness Check

To ensure the reliability of the above-mentioned results, we further used the alternative explanatory variable of shopping online for an additional robustness check. The ordered probit model and IV-ordered probit regression model were employed. The results of the robustness check are shown in Table 4, which suggests that shopping online is positively and significantly associated with homosexuality inclusion in ordered probit (β = 0.074, *p* < 0.01) and IV-ordered probit models (β = 0.224, *p* < 0.01). The abovementioned results regarding the alternative explanatory variable again verify hypothesis 1, and the regression results are consistent across different models. Thus, the results of this paper are robust.

**Table 4:** Robustness check (*N* = 4,037).

**Variable**	**Ordered probit**	**IV-ordered probit**
Shopping online	0.074^***^	0.224^***^
	(0.021)	(0.057)
Gender	−0.071^*^	−0.031
	(0.037)	(0.040)
Education	0.022^***^	0.009
	(0.008)	(0.009)
Age	0.004^**^	0.009^***^
	(0.002)	(0.003)
Household registration	0.092^**^	0.120^**^
	(0.047)	(0.048)
Religion	0.056	0.037
	(0.062)	(0.062)
Political status	−0.053	−0.057
	(0.061)	(0.061)
Education of father	−0.008	−0.010^*^
	(0.005)	(0.005)
Education of mother	0.010	0.006
	(0.006)	(0.006)
Logarithm of income	−0.018	−0.034^*^
	(0.018)	(0.019)
Marital status	−0.167^**^	−0.167^**^
	(0.071)	(0.071)
Social trust	−0.024	−0.010
	(0.024)	(0.024)
Regional effect	Yes	Yes
Pseudo *R*^2^	0.009	-
atanhrho_12	-	−0.171^***^
	-	(0.063)
Observations	4,037	

*Standard errors in parentheses*;

*** *p < 0.01*,

** *p < 0.05*,

* *p < 0.1*.

### Functional Mechanism Analysis

After verifying the positive relationship between of internet-based social media use and homosexuality inclusion, the potential channel through which internet-based social media use was associated with homosexuality inclusion of China was tested. In this paper, the mediation effect of number of LGBT friends was considered.

Table 5 shows the results of bootstrap replication mediation test. Specifically, the results of bootstrapping 500 and 1,000 times both demonstrate that the indirect effects of the number of LGBT friends (indirect effect = 0.018, *p* < 0.01) are significant, and none of the corresponding 95% CIs overlap with zero. The intermediary role that number of LGBT friends plays in the overall relationship between internet-based social media and individuals' homosexuality inclusion is verified. Thus, hypothesis 2 in our study is maintained.

**Table 5:** Mechanism analysis of Bootstrap replications.

**Times**	**Model pathway**	**Estimated effect**	**Percentile 95%CI**	
			**BootLLCI**	**BootULCI**
	**Indirect effect**			
Bootstrap 500 times	Internet-based social media use → Number of LGBT friends → Homosexuality inclusion	0.0180^***^	0.0082	0.0268
		(0.0046)		
	**Indirect effect**			
Bootstrap 1,000 times	Internet-based social media use → Number of LGBT friends → Homosexuality inclusion	0.0180^***^	0.0087	0.0274
		(0.0046)		

*Bootstrap standard error in parentheses; BootLLCI is the Lower Bound and the BootULCI is the Upper Bound*.

*** *p < 0.01. Control variables are controlled in the process of Bootstrap replications*.

## Discussion

The current study explored the relationship between the usage of internet-based social media and Chinese people's attitudes toward homosexuality. The results of descriptive analysis suggested that nearly half of the respondents (49.12%) showed a “somewhat reject” attitude toward homosexuality, and 17.81% of respondents held a “strongly reject” attitude. In 2013, Li and Zheng (2013) conducted a national sampling survey involving 400 Chinese respondents on the public's attitude toward homosexuality, and the results of the investigation showed that nearly 70% of the participants considered homosexuality to be a wrong behavior. Consistent with the survey results from nearly a decade ago, the descriptive analysis results in this study indicate that homosexuality is still a long way from being widely accepted by the public in today's China.

The regression results of the ordered probit benchmark model and the IV-ordered probit model presented a significantly positive correlation between the usage frequency of internet-based social media and individuals' homosexuality inclusion. Prior studies have extensively discussed the influence of personal characteristics and cultural context on individuals' homosexuality inclusion (Adamczyk and Cheng, 2015; Berggren and Nilsson, 2016). In this study, we paid attention the positive influencing factors of technological development on individuals' homosexuality inclusion. As the reciprocal process provided by internet-based social media that involves two-way conversations between information receivers and providers media increases, the social media users' inclusion and acceptance of homosexuality increases. Moreover, compared with traditional mainstream media which generally bans the figures and topics related to homosexuality, the internet-based social media provides a reference group who hold acceptable and inclusion attitude toward homosexuality, and offers opportunities for interacting and making friends with LGBT users. Thus, the frequently usage of internet-based social media ameliorates the hostile of public toward homosexuality.

The results of control variable tests showed that respondents' education and their mothers' education were positively and significantly associated with individual homosexuality inclusion. Many studies have argued that education facilitates the enhancement of an individual's critical thinking, and high levels of education attainment ultimately led to less prejudice of concepts that deviate from traditional norms (Campbell and Horowitz, 2016; Roi and Mandemakers, 2018). Thus, our estimated results of education were consistent with those of the existing literature. Furthermore, as mothers are the primary caregivers of families in Chinese tradition, children's attitudes toward society are affected by mothers to a large extent (Xu and Huang, 2018). This result is consistent with that of a study that focused on intergenerational poverty transmission, which empirically validated the idea that a mother's education had a more positive and significant effect on a child's income than a fathers' (Yang et al., 2021b).

The heterogeneity analysis showed that the homosexuality inclusion of respondents with higher income, respondents of the female gender, and respondents located in eastern region of China were more evidently associated with internet-based social media use. As the prior studies have revealed, income is a vital financial resource to support people in acquiring education and understanding the colorful world firsthand, so people with higher income may be more well-educated, open-minded, and show more inclusion for homosexuality (Carvacho et al., 2013). Similarly, compared with the central and western regions, the eastern region of China has been found to constantly occupy a leading place in economics, and regional economic development is one of the most important determinants of people's income levels.^2^ As such, we found that compared to the central and western regions, the homosexuality inclusion of respondents in the eastern region was more easily and evidently related to internet-based social media use. Moreover, in our sample, females tend to have a favorable attitude toward homosexuality. On the one hand, with women's growing awareness of sexual equality, women start to enjoy the pornographic consumption of men (Wu et al., 2018). On the other hand, compared to men, women may be more aware of the survival difficulties of the homosexual individuals running counter to mainstream values. Therefore, women may be more easily affected by the positive effect of internet-based social media use on homosexuality inclusion than men.

The findings of this paper have some theoretical implications. First, this paper presents the association between internet-based social media use and people's perception and behavior, as well as enriching knowledge of the relationship between information technology and social attitudes toward homosexuality in the Chinese context. The high-profile economic effects of information technology have been extensively explored, but the social effect of information technology development had been ignored to a certain extent (Liu et al., 2020). Therefore, the results in this paper also provide new evidence for the social effects of information technology development, especially in the field of sexual psychology. Second, we regarded internet-based social media as a principal consideration, including by exploring the mechanism through which internet-based social media use theoretically and empirically is associated with homosexuality inclusion and by revealing the heterogeneous effects of internet-based social media use on people's homosexuality inclusion *via* levels of income, gender, and region.

In addition to the above-mentioned contributions, this paper had some limitations we recommend improving in future research. First, homosexuality-related topics are still sensitive in today's Chinese context, and this was the first time that the CLDS asked respondents about their views on homosexuality. Although the dataset used in this paper comprised the latest cross-sectional data and we employed an alternative variable to test the robustness of the results, we faced the problem of missing information on the counterfactual. In other words, we cannot see from the available data and analysis what the attitudes of individuals toward homosexuality were before they used social media and how this attitude changed with the use of internet-based social media. In the future, if data are available, researchers can consider the use of panel data to overcome this shortcoming.

Second, the CLDS considered the homosexuality, gambling, and usurious loan in a same category, which might have negatively affected the participants' answers (this categorization of the CLDS might deserve criticism in itself). Thus, this categorization is another limitation of the current study.

Third, although we have reported on an influential mechanism through which internet-based social media use affects homosexuality inclusion, future research can further explore the potential channels through which the usage of internet-based social media has a positive association with people's homosexuality inclusion.

## Conclusion

We explored the relationship between internet-based social media use and Chinese people's attitudes toward homosexuality. First, by using the nationally representative dataset of the CLDS with ordered probit and IV-ordered probit models, we found that the usage of internet-based social media positively and significantly affects Chinese people's homosexuality inclusion. People with higher use frequencies of internet-based social media tend to show a higher homosexuality inclusion compared to those with lower use frequencies of internet-based social media. Furthermore, the homosexuality inclusion of respondents with higher income, female respondents, and respondents located in the eastern region of China was found to be more evidently associated with internet-based social media use. The results of bootstrap replication mediation test indicated the number of LGBT friends mediated the relationship between individual usage of internet-based social media and homosexuality inclusion.

## Data Availability Statement

The data analyzed in this study are available from the corresponding author on reasonable request.

## Author Contributions

YJ proposed the idea of this paper, developed the method, wrote the theoretical analysis, results, discussion, and conclusions. FY gave guidance in the theory, as well modified and edited the whole manuscript. Both authors have read and agreed to the published version of the manuscript.

## Conflict of Interest

The authors declare that the research was conducted in the absence of any commercial or financial relationships that could be construed as a potential conflict of interest.

## Publisher's Note

All claims expressed in this article are solely those of the authors and do not necessarily represent those of their affiliated organizations, or those of the publisher, the editors and the reviewers. Any product that may be evaluated in this article, or claim that may be made by its manufacturer, is not guaranteed or endorsed by the publisher.

## Footnotes

^1^US$1 = 6.405 yuan (Accessed October 31, 2021).

^2^Comparison of GDP in different regions of China. Available online at: https://baijiahao.baidu.com/s?id=1660311082536759882&wfr=spider&for=pc (Accessed August 19, 2021).

## References

[B1] Adamczyk A. Cheng Y. A. (2015). Explaining attitudes about homosexuality in Confucian and non-Confucian nations: is there a ‘cultural' influence? Soc. Sci. Res. 51, 276–289. 10.1016/j.ssresearch.2014.10.002 25769867

[B2] Berggren N. Nilsson T. (2016). Tolerance in the United States: does economic freedom transform racial, religious, political and sexual attitudes? Eur. J. Polit. Econ. 45, 53–70. 10.1016/j.ejpoleco.2016.06.001

[B3] Blued (2018). Available online at: https://www.blued.cn/aboutus.html#intro (accessed August 19, 2021).

[B4] Bruning P. F. Alge B. J. Lin H. C. (2020). Social networks and social media: understanding and managing influence vulnerability in a connected society. Bus. Horizons 63, 749–761. 10.1016/j.bushor.2020.07.007

[B5] Burki T. (2017). Health and rights challenges for China's LGBT community. Lancet 389, 1286. 10.1016/S0140-6736(17)30837-1 28379143

[B6] Campbell C. Horowitz J. (2016). Does college influence sociopolitical attitudes? Sociol. Educ. 89, 40–58. 10.1177/0038040715617224

[B7] Carvacho H. Zick A. Haye A. González R. Manzi J. Kocik C.. (2013). On the relation between social class and prejudice: the roles of education, income, and ideological attitudes. Eur. J. Soc. Psychol. 43, 272–285. 10.1002/ejsp.1961

[B8] Chauvin J. P. Glaeser E. Ma Y. Tobio K. (2016). What is different about urbanization in rich and poor countries? Cities in Brazil, China, India and the United States. J. Urban Econ. 98, 17–49. 10.1016/j.jue.2016.05.003

[B9] Cheng Y. A. Wu F. C. F. Adamczyk A. (2016). Changing attitudes toward homosexuality in Taiwan, 1995–2012. Chin. Sociol. Rev. 48, 317–345. 10.1080/21620555.2016.1199257

[B10] Chi X. Hawk S. T. (2016). Attitudes toward same-sex attraction and behavior among Chinese university students: tendencies, correlates, and gender differences. Front. Psychol. 7, 1592. 10.3389/fpsyg.2016.01592 27790184 PMC5062029

[B11] China Internet Network Information Center (CNNIC) (2022). The 49th Statistical Report on China's Internet Development. Available online at: http://www.cnnic.net.cn/hlwfzyj/hlwxzbg/hlwtjbg/202202/P020220407403488048001.pdf (accessed April 10, 2022).

[B12] Choi S. Y. P. Luo M. (2016). Performative family: homosexuality, marriage and intergenerational dynamics in China. Br. J. Sociol. 67, 260–280. 10.1111/1468-4446.12196 27206789

[B13] Chow J. Y. Konda K. A. Calvo G. M. Klausner J. D. Caceres C. F. (2017). Demographics, behaviors, and sexual health characteristics of high-risk men who have sex with men and transgender women who use social media to meet sex partners in Lima, Peru. Sex. Trans. Dis. 44, 143–148. 10.1097/OLQ.0000000000000566 28178111 PMC6879100

[B14] Feng Y. Lou C. Gao E. Tu X. Cheng Y. Emerson M. R.. (2012). Adolescents' and young adults' perception of homosexuality and related factors in three Asian cities. J. Adolesc. Health 50, S52–S60. 10.1016/j.jadohealth.2011.12.008 22340857 PMC4165856

[B15] Fisman R. Svensson J. (2007). Are corruption and taxation really harmful to growth? Firm level evidence. J. Dev. Econ. 83, 63–75. 10.1016/j.jdeveco.2005.09.009

[B16] Goodman M. B. Moradi B. (2008). Attitudes and behaviors toward lesbian and gay persons: critical correlates and mediated relations. J. Couns. Psychol. 55, 371–384. 10.1037/0022-0167.55.3.371

[B17] Hu K. Li X. (2019). The Effects of media use and traditional gender role beliefs on tolerance of homosexuality in China. Chin. Sociol. Rev. 1, 147–172. 10.1080/21620555.2019.1595567

[B18] Hu S. Pattatucci A. M. L. Patterson C. Li L. Fulker D. W. Cherny S. S.. (1995). Linkage between sexual orientation and chromosome Xq28 in males but not in females. Nat. Genet. 11, 248–256. 10.1038/ng1195-248 7581447

[B19] Joslyn M. R. Haider-Markel D. P. (2016). Genetic attributions, immutability, and stereotypical judgments: an analysis of homosexuality. Soc. Sci. Quart. 97, 376–390. 10.1111/ssqu.12263

[B20] Kong T. Z. He Y. N. Auerbach R. P. McWhinnie C. M. Xiao J. (2015). Rumination and depression in Chinese university students: the mediating role of overgeneral autobiographical memory. Pers. Individ. Diff. 77, 221–224. 10.1016/j.paid.2014.09.035 25977594 PMC4428603

[B21] Li C. Y. Kent M. L. (2021). Explorations on mediated communication and beyond: toward a theory of social media. Public Relat. Rev. 47, 102112. 10.1016/j.pubrev.2021.102112

[B22] Li H. Holroyd E. Lau J. T. F. (2010). Negotiating homosexual identities: the experiences of men who have sex with men in Guangzhou. Cult. Health Sex. 12, 401–414. 10.1080/13691050903551721 20162481

[B23] Li Y. H. Zheng H. X. (2013). Public attitude towards homosexuality and its influencing factors. J. South China Normal Univ. (*Soc. Sci. Ed*.) 6, 31–36. (In Chinese). 10.3969/j.issn.1671-3060.2013.12.015

[B24] Li Z. Gao X. (2021). Makers' relationship network, knowledge acquisition and innovation performance: an empirical analysis from China. Technol. Soc. 66, 101684. 10.1016/j.techsoc.2021.101684

[B25] Liang Z. R. Huang Y. T. Chen Y. C. Chan L. S. (2022). “Pattern Matters”: latent class analysis of internet use and users' attitudes toward homosexuality in China. Sex. Res. Soc. Policy. 10.1007/s13178-021-00680-w

[B26] Lin K. Button D. M. Su M. Chen S. (2016). Chinese college students' attitudes toward homosexuality: exploring the effects of traditional culture and modernizing factors. Sex. Res. Soc. Policy 13, 158–172. 10.1007/s13178-016-0223-3

[B27] Liu J. D. Cheng M. W. Wei X. Y. Yu N. N. (2020). The Internet-driven sexual revolution in China. Technol. Forec. Soc. Change 153, 119911. 10.1016/j.techfore.2020.119911

[B28] Martínez-López F. J. Li Y. Liu H. Feng C. (2020). Do safe buy buttons and integrated path-to-purchase on social platforms improve users' shopping-related responses? Electr. Comm. Res. Appl. 39, 100913. 10.1016/j.elerap.2019.100913

[B29] Nathan M. L. Ormond K. E. Dial C. M. Gamma A. Lunn M. R. (2018). Genetic counselors' and genetic counseling students' implicit and explicit attitudes toward homosexuality. J. Genet. Couns. 28, 91–101. 10.1007/s10897-018-0295-8 30168102

[B30] National Bureau of Statistics (2011). Regional Division. Available online at: http://www.stats.gov.cn/ztjc/zthd/sjtjr/dejtjkfr/tjkp/201106/t20110613_71947.htm (accessed March 31, 2022).

[B31] Roi C. L. Mandemakers J. J. (2018). Acceptance of homosexuality through education? Investigating the role of education, family background and individual characteristics in the United Kingdom. Soc. Sci. Res. 71, 109–128. 10.1016/j.ssresearch.2017.12.006 29514752

[B32] Sautter J. M. Tippett R. M. Morgan S. P. (2010). The social demography of Internet dating in the United States. Soc. Sci. Quart. 91, 554–575. 10.1111/j.1540-6237.2010.00707.x24232583

[B33] Shao L. (2018). The dilemma of criticism: disentangling the determinants of media censorship in China. J. East Asian Stud. 18, 279–297. 10.1017/jea.2018.19

[B34] Stroud N. J. (2007). Media use and political predispositions: revisiting the concept of selective exposure. Polit. Behav. 30, 341–366. 10.1007/s11109-007-9050-9

[B35] Tu J. Lee T. (2014). The effects of media usage and interpersonal contacts on the stereotyping of lesbians and gay men in China. J. Homosexual. 61, 980–1002. 10.1080/00918369.2014.871904 24325259

[B36] Wang H. Cai T. Mou Y. Shi F. (2018). Traditional resources, internet resources, and youth online political participation: the resource theory revisited in the Chinese context. Chin. Sociol. Rev. 50, 115–136. 10.1080/21620555.2017.1341813

[B37] Wang Y. Hu Z. Peng K. Xin Y. Yang Y. Drescher J.. (2019). Discrimination against LGBT populations in China. Lancet Public Health 4, e440–e441. 10.1016/S2468-2667(19)30153-7 31493836

[B38] Wu C. (2004). Homoerotic Sensibilities in Late Imperial China. Curzon, London: Routledge.

[B39] Wu Y. Mou Y. Wang Y. Atkind D. (2018). Exploring the de-stigmatizing effect of social media on homosexuality in China: an interpersonal-mediated contact versus parasocial-mediated contact perspective. Asian J. Commun. 28, 20–37. 10.1080/01292986.2017.1324500

[B40] Xie Y. Peng M. (2017). Attitudes towards homosexuality in China: exploring the effects of religion, modernizing factors, and traditional culture. J. Homosexual. 65, 1758–1787. 10.1080/00918369.2017.1386025 28956732

[B41] Xu J. Huang Y. (2018). Gender identity, marriage and labor behavior within households. Econ. Res. J. 4, 135–150. (In Chinese)

[B42] Yang F. Jiang Y. (2020). Heterogeneous influences of social support on physical and mental health: Evidence from China. Int. J. Environ. Res. Public Health 17, 6838. 10.3390/ijerph17186838 32962140 PMC7558190

[B43] Yang F. Jiang Y. (2022). Adolescent self-control and individual physical and mental health in adulthood: a Chinese study. Front. Psychol. 13, 850192. 10.3389/fpsyg.2022.850192 35444588 PMC9013772

[B44] Yang F. Jiang Y. Paudel K. P. (2021a). Impact of credit constraints from formal financial institutions on rural residents' health in China. Healthcare 9, 6. 10.3390/healthcare9010006 33374650 PMC7822418

[B45] Yang F. Paudel K. P. Jiang Y. (2022). Impact of self-control on individual income: evidence from China. Econ. Res. Ekonom. IstraŽivanja. 10.1080/1331677X.2022.2048190

[B46] Yang F. Paudel K. P. Jiang Y. (2021b). Like parents, like children? Intergenerational poverty transmission in China. J. Asia Pacific Econ. 10.1080/13547860.2021.1960615

[B47] Yen C. F. Pan S. M. Hou S. Y. Liu H. C. Wu S. J. Yang W. C.. (2007). Attitudes toward gay men and lesbians and related factors among nurses in Southern Taiwan. Public Health 121, 73–79. 10.1016/j.puhe.2006.08.013 17166534

[B48] Zhang B. C. (1994). Homosexuality. Shandong: Shandong Science and Technology Press (In Chinese).

[B49] Zhao P. J. Yu Z. (2020). Investigating mobility in rural areas of China: features, equity, and factors. Trans. Policy 94, 66–77. 10.1016/j.tranpol.2020.05.008

[B50] Zheng Q. Guo Y. Wang Z. Andrasik F. Kuang Z. Li J.. (2022). Exploring Weibo users' attitudes toward lesbians and gays in Mainland China: a natural language processing and machine learning approach. Comput. Hum. Behav. 127, 107021. 10.1016/j.chb.2021.107021

